# Malnutrition Screening and Assessment in the Cancer Care Ambulatory Setting: Mortality Predictability and Validity of the Patient-Generated Subjective Global Assessment Short form (PG-SGA SF) and the GLIM Criteria

**DOI:** 10.3390/nu12082287

**Published:** 2020-07-30

**Authors:** Lynette M. De Groot, Gahee Lee, Antoinette Ackerie, Barbara S. van der Meij

**Affiliations:** 1 Dietetics and Foodservices, Mater Health, Brisbane, 4101 QLD, Australia; lynette.degroot@gmail.com (L.M.D.G.); antoinette.ackerie@mater.org.au (A.A.); 2 Mater Research Institute, University of Queensland, Brisbane, 4101 QLD, Australia; 3 Bond University Nutrition and Dietetics Research Group, Faculty of Health Sciences and Medicine, Bond University, Gold Coast, 4226 QLD, Australia; gahee.lee@student.bond.edu.au

**Keywords:** malnutrition, cancer, handgrip strength, nutrition assessment

## Abstract

Background: A valid malnutrition screening tool (MST) is essential to provide timely nutrition support in ambulatory cancer care settings. The aim of this study is to investigate the validity of the Patient-Generated Subjective Global Assessment Short Form (PG-SGA SF) and the new Global Leadership Initiative on Malnutrition (GLIM) criteria as compared to the reference standard, the Patient-Generated Subjective Global Assessment (PG-SGA). Methods: Cross-sectional observational study including 246 adult ambulatory patients with cancer receiving in-chair intravenous treatment at a cancer care centre in Australia. Anthropometrics, handgrip strength and patient descriptive data were assessed. Nutritional risk was identified using MST and PG-SGA SF, nutritional status using PG-SGA and GLIM. Sensitivity (Se), specificity (Sp), positive and negative predictive values and kappa (k) were analysed. Associations between malnutrition and 1-year mortality were investigated by Cox survival analyses. Results: A PG-SGA SF cut-off score ≥5 had the highest agreement when compared with the PG-SGA (Se: 89%, Sp: 80%, k = 0.49, moderate agreement). Malnutrition risk (PG-SGA SF ≥ 5) was 31% vs. 24% (MST). For malnutrition according to GLIM, the Se was 76% and Sp was 73% (k = 0.32, fair agreement) when compared to PG-SGA. The addition of handgrip strength to PG-SGA SF or GLIM did not improve Se, Sp or agreement. Of 100 patients who provided feedback, 97% of patients found the PG-SGA SF questions easy to understand, and 81% reported that it did not take too long to complete. PG-SGA SF ≥ 5 and severe malnutrition by GLIM were associated with 1-year mortality risk. Conclusions: The PG-SGA SF and GLIM criteria are accurate, sensitive and specific malnutrition screening and assessment tools in the ambulatory cancer care setting. The addition of handgrip strength tests did not improve the recognition of malnutrition or mortality risk.

## 1. Introduction

In 2019, it is estimated that almost 145,000 new cases of cancer will be diagnosed in Australia [1]. Throughout their cancer journey, around 30–90% of patients experience malnutrition [2,3]. This is concerning, as malnutrition is associated with reduced treatment effectiveness [4,5,6], functional status [4,6], quality of life [4,7] and survival [4,5,6,8]. Research suggests that early nutrition intervention may prevent nutritional deterioration in patients with cancer [9,10,11] and as such it is therefore recommended that regular nutrition screening to facilitate timely nutrition care occurs for all patients undergoing cancer treatment [12].

Patients with cancer often receive treatment in outpatient or ambulatory day care settings. To identify malnutrition risk in these patients, it is essential to choose an efficient nutritional screening tool suitable for ambulatory cancer patients. We know that many screening tools can lack clinical information, sensitivity, specificity and/or are not usable and applicable for busy cancer care centres in which there are high turnover rates of outpatients and limited human resources [13].

A number of malnutrition screening tools exist, with the Malnutrition Screening Tool (MST) being the most commonly used in Australia. The MST has a good validity, sensitivity and specificity to identify malnutrition assessed using the Patient-Generated Subjective Global Assessment (PG-SGA) in ambulatory cancer care patients. The PG-SGA is widely used in clinical practice for assessing nutritional status of cancer patients. The Patient-Generated Subjective Global Assessment Short Form (PG-SGA SF), a component of the full PG-SGA, has recently been receiving new attention as a valid screening tool for nutritional risk [14] and meets the professional standard when compared to the PG-SGA [15,16]. The PG-SGA SF retains the patient completed section of weight history, food intake, nutrition symptoms and physical function, and foregoes the remainder. The optimal cut-off value or score to determine malnutrition risk according to the PG-SGA SF differs in the research and depends on the purpose or objectives of the team using the instrument in clinical practice [17]. Since it is designed to be completed by the patient, it is relatively simple to complete, and it reduces time and additional burden to healthcare professionals [15]. Patient-led screening may improve patient autonomy and aligns with the international focus on patient-centred care. Recently, patient-led screening with the MST was found to be reliable and well accepted by patients attending an ambulatory cancer care setting [18].

Interestingly, to standardize the clinical practice of malnutrition diagnosis in clinical settings, the Global Leadership Initiative on Malnutrition (GLIM) recently proposed diagnostic criteria for malnutrition. The GLIM criteria are objective, global and based on consensus. The GLIM includes a combination of phenotypic (percentage weight loss, low body mass index, reduced muscle mass) and etiologic (reduced food intake or assimilation, acute or chronic inflammation) criteria for the diagnosis of malnutrition. The GLIM criteria have not yet been validated for identifying malnutrition in an ambulatory oncology population, nor its predictive value regarding survival in these patients. There is well established evidence of the PG-SGA’s ability to predict clinical outcomes including survival with well-nourished oncology patients having longer survival duration than malnourished patients [14,15].

It has also been shown that handgrip strength is reduced in cancer patients with malnutrition [19]. Handgrip strength has been shown to be a prognostic marker and is positively associated with survival duration, for instance, in older patients with cancer [20,21]. As the aim of nutritional therapy is to restore muscle mass and muscle strength, handgrip strength can serve as an additional parameter to improve the recognition of malnutrition risk or malnutrition. Assessment of muscle function using grip strength is recommended as a supportive measure in the GLIM consensus [22].

Therefore, the aim of this study is to (1) evaluate the agreement, sensitivity and specificity of the PG-SGA SF and GLIM criteria to reference standard of PG-SGA, (2) investigate the added value of handgrip strength test to PG-SGA SF and GLIM to recognise malnutrition risk or malnutrition, (3) evaluate the patient experience and ease of completion of the patient-completed PG-SGA SF and (4) investigate the ability of these tools to predict patient outcomes such as mortality and hospitalisation and whether any tool is superior in this ability.

## 2. Materials and Methods

We conducted a cross-sectional, observational study at the Mater Cancer Care Centre (MCCC) in Brisbane, QLD, Australia over one week in May 2018 and repeated over one week in March 2019. Patient outcome data were collected 12 months post initial data collection. The protocol received was approved as low risk research by the Human Research Ethics Committee of Mater Misericordiae Limited (HREC/18/MHS/101).

### 2.1. Participants

Patients with cancer aged 18 years or older were included if they were receiving in-chair intravenous treatment at MCCC. People who were receiving treatment for a benign condition (e.g., rheumatoid arthritis), receiving a blood transfusion only, waiting on hospital admission, or who declined to take part in this study were excluded from participation.

### 2.2. PG-SGA SF

Patients who consented to the study were asked to complete a paper-based PG-SGA SF and received basic verbal instructions (such as ‘tick the box that is applicable’) by trained staff. The PG-SGA SF consists of four boxes: box 1, questions regarding body weight (scored 0–5); box 2, food intake (score 0–4); box 3, symptoms affecting oral food intake (scored 0–23); and box 4, regarding activities and function. Based on findings from a previous Australian study [16], box 4 questions were excluded from the PG-SGA SF used in this study. Upon completion, the scores of boxes 1, 2 and 3 were totalled by a dietitian.

A short questionnaire to assess ease of completion and time taken to complete the PG-SGA SF was provided to each patient in 2019. The questionnaire was based on previously developed tools [18] and included four questions regarding clarity and understanding of the tool, and time taken to complete the tool.

### 2.3. MST and PG-SGA

In line with established guidelines [12], all participants were screened for malnutrition using the MST. The MST includes two questions about recent unintentional weight loss and reduced appetite affecting dietary intake [23] with answers generating a numerical score between 0 and 5. Patients with an MST score ≥2 were classified as ‘at risk of malnutrition’ and were assessed by dietitians using the PG-SGA to determine their degree of malnutrition. The PG-SGA classifies patients into three categories: (A) well-nourished; (B) moderately malnourished; or (C) severely malnourished. For data analysis purposes, patients with MST <2 were assumed as well nourished (PG-SGA A).

### 2.4. GLIM Criteria

To diagnose malnutrition using the GLIM criteria, weight changes within six months (%) and body mass index (BMI) were calculated using patients’ weight history and height (Table 1). Based on the GLIM criteria, participants who had a combination of at least one phenotypic criterion (weight loss and/or low BMI for age) and one etiologic criterion (disease burden and inflammatory condition of cancer) were categorised as malnourished. The remaining participants were categorised as well-nourished.

### 2.5. Handgrip Strength (HGS)

Handgrip strength tests were performed in the dominant hand using a hydraulic hand dynamometer (Jamar Plus+, Performance Health Supply Inc., Sutton-in-Ashfield, UK). The patient performed the test in a seated position, with the shoulder abducted and neutrally rotated, elbow flexed at 90 degrees, forearm and wrist in a neutral position. Patients were instructed to perform three maximal isometric contractions. Patients took brief pauses between measurements. The maximal value was recorded to the nearest 0.1 kg, and was used to compare with the 10th percentile of age- and gender-dependent reference values [24]. If patients were unable to perform handgrip strength with their dominant hand, the non-dominant hand was used.

### 2.6. Patient Outcomes

Patient outcome measures were collected 12 months post initial study date and were obtained from the patient electronic medical record. This included mortality, number of hospital admissions during the 12-month period and length of stay of hospital admissions.

### 2.7. Statistical Analysis

Data analysis was completed using IBM SPSS software V.25.0 for Windows. A significance level of 5% was applied to detect statistical significance. For normally distributed continuous variables, independent t-tests were applied to compare means between malnutrition categories. PG-SGA SF values from 1 to 7 were each analysed to determine optimal cut-off values to determine malnutrition risk for this population. In order to compare the PG-SGA SF and GLIM with the reference instrument (PG-SGA), sensitivity, specificity and positive and negative predictive values for the PG-SGA SF and GLIM against PG-SGA were calculated and receiver operating characteristic (ROC) curves were generated. Kappa coefficient was assessed to investigate the rate of agreement between PG-SGA SF or GLIM and PG-SGA. To determine the validity of the PG-SGA SF, the professional standard 80% for sensitivity and 60% for specificity were determined based on the literature [25,26]. The kappa coefficient was interpreted based on the literature [27,28] as follows: <0 as poor agreement; 0.01–0.20 as slight agreement; 0.21–0.40 as fair agreement; 0.41–0.60 as moderate agreement; 0.61–0.80 as substantial agreement; and 0.81–1.00 as almost perfect agreement. The professional standard for kappa was set to >0.60 [29]. Cox proportional hazards analyses were performed to determine the association between malnutrition diagnoses and mortality 1 year after the audit date. Univariate as well as multivariate analyses adjusted for gender, age (≤65 years vs. >65 years), obesity (BMI ≥ 30 kg/m^2^) and diagnosis (breast cancer vs. other types of cancer) were performed on the basis of available literature. The association between malnutrition diagnoses and hospital admissions (Y/N) and length of stay was investigated by logistic and linear regression analysis, respectively (both univariate and multivariate, adjusted for similar confounders).

## 3. Results

Out of 275 patients that were eligible, a total of 246 patients consented to participate in the study. The mean age was 61.9 ± 13.1 years, and 182 (74%) patients were female. The most common cancer diagnoses were breast (45%), gynaecological (13%) and colorectal (11%) (Table 2).

### 3.1. Validity of Malnutrition Tools/Instruments

The MST had a sensitivity of 100% and a specificity of 90% (kappa: 0.737) when compared to the reference tool PG-SGA.

The sensitivity and specificity for the different cut-off scores and percentiles of PG-SGA SF are depicted in Table 3. The sensitivity was the highest when applying a cut-off score ≥3 for PG-SGA SF, however cut-off scores ≥3, ≥4 and ≥5 all fulfilled the criteria deemed acceptable for validity. A PG-SGA SF cut-off score ≥5 had the highest agreement and deemed most suitable when compared with the reference PG-SGA, with a sensitivity of 89%, a specificity of 80% and a ‘moderate agreement’ (k = 0.493).

When compared to the reference standard PG-SGA, malnutrition according to the GLIM criteria had a sensitivity of 76%, specificity of 73% and a ‘fair agreement’ (k = 0.323). When adding handgrip strength < 10th percentile of reference values, sensitivity and specificity for both PG-SGA SF and GLIM declined to around 20% and 95–96%, respectively and kappa declined to approximately 0.2 (poor to slight agreement).

### 3.2. Malnutrition Risk and Malnutrition

According to the MST, 60 patients (24%) were identified as at risk of malnutrition compared to 71 (31%) patients with PG-SGA SF ≥ 5 (Table 4). There were 32 (14%) patients with a handgrip strength (HGS) cut-off score of <10th percentile. When handgrip strength < 10th percentile of reference values was added to PG-SGA SF ≥ 5 the number of patients identified as at risk of malnutrition decreased to 14 (6%).

According to the PG-SGA assessed by the dietitian, the number of patients who identified as malnourished was 39 (16%), with 33 (13%) of those moderately (PG-SGA B) malnourished. According to the GLIM criteria for the diagnosis of malnutrition; 77 (35%) out of 220 patients were identified as malnourished (moderately, severely, or both). When handgrip strength was added to the GLIM criteria (HGS < 10th percentile), malnutrition prevalence was reduced to 7% (*n* = 14).

Malnutrition according to the PG-SGA was identified across all BMI categories, with the lowest percentage of malnutrition in obese patients. Cancer types with the highest rates of malnutrition were respiratory (*n* = 21, 29%), haematology (*n* = 21, 24%) and colorectal cancer (*n* = 27, 22%). In patients receiving chemotherapy agents with a moderate or high emetogenicity risk, more patients were malnourished (24%) compared to patients receiving low emetogenicity risk agents (10%) (*p* = 0.002). The most frequently reported nutrition impact symptom in the malnourished group was ‘no appetite’ (*n* = 23, 59%), followed by ‘things taste funny or have no taste’ (*n* = 18, 46%) and ‘fatigue’ (*n* = 12, 31%).

A reduced food intake in the past month was reported by 66 patients (29.5%). In malnourished patients, a food intake less than usual was reported by 80% (*n* = 28), compared to only 20% (*n* = 38) in well-nourished patients (*p* < 0.001).

### 3.3. Patient Experience

Table 5 displays data on patients’ experiences with self-completing the PG-SGA SF during the 2019 audit. A 95% completion rate was achieved (100 out of 105 questionnaires completed). According to the questionnaire responses, 98% of patients indicated that the instructions to complete the tool were clear (*n* = 101), and most found the questions easy to understand (*n* = 101, 97%). Furthermore, 81% of patients thought the questions did not take too long to complete (*n* = 100) with most reporting it took five minutes or less to complete the PG-SGA SF (*n* = 100, 97%).

### 3.4. Patient Outcomes

Multivariate analyses showed that malnutrition according to the GLIM criteria was associated with a 2-fold increased 1-year mortality risk, and a 4-fold increased risk when adding handgrip strength lower than the 10th percentile of reference value as a muscle strength parameter. However, these associations were borderline statistically significant (Table 6). Severe malnutrition according to the GLIM criteria was associated with increased mortality risk (HR 2.9, *p* = 0.019); this was not the case for moderate malnutrition according to GLIM, or for moderate or severe malnutrition (GLIM) when adding handgrip strength < 10th percentile of reference values.

Malnutrition risk according to the PG-SGA SF (both with cut-off ≥3 and ≥5) was associated with a 1.6 or 3.5 increased mortality risk, respectively (*p* = 0.006 and *p* = 0.004). When including handgrip strength, the PG-SGA SF was not associated with 1-year mortality risk.

The reference tools were both associated with 1-year mortality risk: hazard ratio (HR) 3.4 (*p* = 0.004) for MST ≥ 2, and HR 10.4 (*p* < 0.001) for PG-SGA B and PG-SGA C. No associations were observed between number of hospital admissions or total length of stay in hospital during 1-year post initial assessment.

## 4. Discussion

Findings from this study demonstrate that the PG-SGA SF is an accurate, sensitive and specific malnutrition screening tool in the ambulatory cancer care setting. This is consistent with other studies [14,16] when compared to the full PG-SGA.

Our study identified that cut-off scores of ≥3, ≥4 and ≥5 all fulfilled criteria deemed acceptable for validity, however a cut-off score of ≥5 had the highest agreement with the reference standard of malnutrition according to PG-SGA. Optimal cut-off scores differ according to the literature, reporting ≥3 [16] and ≥6 [15] as optimal cut-off scores. This disparity may be due to different administration methods (patient completed vs. clinician completed). In the current study the patient self-completed the PG-SGA SF. This is more comparable to Gabrielson et al., reporting ≥6 as the optimal cut-off [15]. One reason for our lower cut-off score of ≥5 would be our formatting of the PG-SGA SF, which was the removal of the activity box (box 4). This is a study limitation, however, which accounts for the lower cut-off score.

When compared to MST, the PG-SGA SF identified more patients at risk of malnutrition (31% vs. 24%). This could be explained by the inclusion of nutrition impact symptoms in the PG-SGA SF. Nutrition impact symptoms were common in our cohort, with 29% of patients reporting at least one symptom in the last two weeks. The PG-SGA SF provides valuable information on nutrition impact symptoms, which alone succeeded in identifying patients at risk of malnutrition in previous studies [16,30].

It has been hypothesized that identifying nutrition impact symptoms, especially early in the cancer continuum, may facilitate pro-active malnutrition prevention [14]. For example, patients with no significant weight loss reporting several nutrition impact symptoms are at risk of deterioration in nutritional status and quality of life if they do not receive timely intervention.

Previous studies have indicated that patient-led screening is quick and easy for patients to complete [18]. In our study, patients were provided simple instructions to self-complete the tool, with the addition of patient experience questions in year two of the study. This was included to evaluate the usability and acceptability of the patient completed tool within our local setting. Patient completed PG-SGA SF was well accepted in our study with most indicating the questions easy to understand and that it took five minutes or less to complete the tool. This is similar to a previous study in an ambulatory cancer care setting where they found patient-led screening with the MST was reliable and well accepted by patients [18]. The benefit of the PG-SGA SF over the MST is the additional contributing factors associated with poor dietary intakes, enabling the delivery of the most efficient nutrition intervention [6,31,32].

Malnutrition prevalence in our population differed greatly according to which tool was used, with the GLIM criteria identifying two times the number of patients as malnourished compared to the PG-SGA (35% vs. 16%). PG-SGA is a subjective diagnostic tool that is validated, reliable and widely used in the oncology setting [33,34,35]. Whilst there is no gold standard for determining nutritional status, for the purpose of our study we used PG-SGA as a primary reference tool to determine the validity of GLIM criteria for malnutrition. When compared to this reference standard PG-SGA, malnutrition according to the GLIM criteria had a sensitivity of 76%, specificity of 73% and a ‘fair agreement’ (k = 0.323). This falls slightly short of the acceptable professional standard of 80% and 60%. The difference in prevalence rates between PG-SGA and GLIM criteria can be theorised by the time frame of involuntary weight loss and the use of BMI. The PG-SGA classification is based on 1-month weight loss percentage, whereas the GLIM criteria include weight loss up to 6 months and beyond 6 months. This may account for the high prevalence according to GLIM, where patients with a history of weight loss that have stabilised weight in the recent period may be classified as not malnourished according to PG-SGA. Additionally, a low BMI as a phenotypic criterion can classify patients as malnourished according to GLIM, whereas BMI is not used in the PG-SGA. There is substantial variation in the use of low BMI as a phenotypic criterion for diagnosis of malnutrition and further research on this is required [36].

Furthermore, in our study a PG-SGA was only completed on patients who scored MST ≥ 2, with all other patients assumed as PG- SGA A (well nourished), whereas GLIM criteria were assessed for all patients. Finally, the PG-SGA is a subjective diagnostic tool, in contrast, GLIM is an objective measurement which determines patients with malnutrition using objective data and cut-offs, and not by clinician’s or patient’s judgement [22]. This means that the skill and experience of clinicians may impact less on validity and reliability of the assessment when using the GLIM. For these reasons, studies evaluating the validity of a malnutrition tool, should use an objective reference tool rather than a subjective tool. The GLIM initiative targets the priority to adopt global consensus criteria so that malnutrition prevalence interventions and outcomes can be compared throughout the world [22]. The consensus criteria targets adults in clinical settings, although not specifically for a disease state. The GLIM criteria do not entail the robust detail of comprehensive nutrition assessment but provide a malnutrition diagnosis that may be complemented by more comprehensive assessment, for example the PG-SGA, to provide the basis for individualised treatment plans.

Handgrip strength is a non-invasive, quick and easy method to assess peripheral muscle strength and has been proposed as an indicator of muscle wasting and malnutrition [36]. In cancer patients, muscle wasting is particularly frequent and is associated with increased morbidity and mortality [37]. In fact, in a study in patients with advanced-stage cancer, 6-month mortality was higher amongst malnourished as assessed by the SGA (Subjective Global Assessment) and GLIM criteria using HGS (using 5th percentile cut-off) [38]. It should be kept in mind that hand grip strength is an indicator of upper limb strength and muscle function only and despite its apparent predictive potential, it cannot replace assessment of muscle mass by validated body composition techniques (dual energy x-ray absorptiometry (DXA), bioelectrical impedance analysis (BIA), computed tomography (CT), magnetic resonance imaging (MRI)). Muscle strength tools can be used as a supporting proxy [22]. According to our findings, the addition of a handgrip strength test did not improve the recognition of malnutrition risk when combined with PG-SGA SF. In fact, the sensitivity decreased to 21% (specificity 96%), and the agreement with PG-SGA was poor. Furthermore, the addition of a handgrip strength test also did not improve the recognition of malnutrition when combined to the GLIM criteria.

There is well-established evidence of the PG-SGA’s ability to predict clinical outcomes, such as patient survival, postoperative complications, length of stay, quality of life and hospitalisation costs [14]. Given the relatively new nature of the GLIM criteria, there are few studies investigating the predictive ability in a cancer population. Recent studies found that malnutrition according to GLIM criteria with the addition of hand grip strength in hospitalised cancer patients is associated with higher mortality, specifically a 2–3-fold increase in mortality [38,39]. These findings are similar to our results, which confirm that the GLIM criteria are sensitive to identify mortality risk. We note however, that it is difficult to compare findings due to different populations, stages of disease and settings (ambulatory vs. hospitalised), as well as differing hand grip strength references. Thus, more research is necessary to investigate this association.

### 4.1. Limitations

The principal limitation of our study was removal of the activity box (box 4) from the PG-SGA SF and therefore not using the tool as it was designed. This decision was based on findings by Abbott et al., [16] showing that PG-SGA and additive score combinations of the first three boxes (removing box 4) had higher sensitivity than the MST in ambulatory cancer patients.

Another major limitation of our study was that all patients with MST <2 were assumed as well- nourished PG-SGA A. As this study was initially completed as part of the annual malnutrition prevalence auditing, the usual procedure was to progress to PG-SGA only if MST ≥2. To enable analysis of data, all patients with MST <2 were then assumed as PG-SGA A. This may have incorrectly labelled patients as well-nourished when in fact they could have been malnourished.

Additionally, the study was completed over two separate weeks in two separate years with slightly different methods applied. The addition of patient experience questions was only included in the second year which meant that this valuable information was only obtained from 40% of the study population. Other limitations include our sample size of 246 patients, and heterogeneity of diagnoses.

### 4.2. Implications for Practice and Research

It is challenging to implement routine malnutrition screening in ambulatory cancer care settings. Resources tend to be stretched, therefore screening tools should be quick and simple. We found that the PG-SGA SF is a valid and suitable screening tool for cancer patients and is a good alternative to the MST. The addition of a handgrip strength test did not add value to screening or diagnosing malnutrition; it created extra work for clinicians and burden to patients and therefore has not been implemented as standard care in our cancer centre. The benefit of the PG-SGA SF in being patient-led aligns with core patient centred concepts and with engaging patients in the care process, and our data showed a good acceptance and patient experience. PG-SGA SF and the GLIM criteria both predict patient mortality, however, there needs to be more large-scale studies to validate the GLIM criteria and compare to reference assessment tools, and body composition techniques.

## 5. Conclusions

The PG-SGA SF is an accurate, sensitive and specific malnutrition screening tool in the ambulatory cancer care setting. It is patient completed which aligns with patient-centred concepts, and it is well accepted by patients. The addition of a handgrip strength test did not add value, in fact, it reduced the sensitivity in screening or diagnosing malnutrition. The GLIM criteria diagnosed a greater percentage of malnutrition when compared to PG-SGA and the agreement of GLIM to this reference tool is fair. The GLIM criteria should not replace the use of a comprehensive nutrition assessment and should be used in parallel with established and validated tools such as PG-SGA.

## Figures and Tables

**Table 1:** Global Leadership Initiative on Malnutrition (GLIM) phenotypic and etiologic criteria for the diagnosis of malnutrition.

Phenotypic Criteria			Etiologic Criteria	
Weight Loss (%)	Low Body Mass Index (kg/m^2^)	Reduced Muscle Mass ^a^	Reduced Food Intake or Assimilation ^b,c^	Inflammation ^d–f^
>5% within past 6 months or >10% beyond 6 months	<20 if <70 years, or <22 if >70 years Asia: <18.5 if <70 years, or <20 if >70 years	Reduced by validated body composition measuring techniques	≤50% of ER >1 week, or any reduction for >2 weeks, or any chronic GI condition that adversely impacts food assimilation or absorption	Acute disease/injury ^d,f^ or chronic disease-related ^e,f^

ER = energy requirements, GI = gastrointestinal. Requires at least one phenotypic criterion and one etiologic criterion for diagnosis of malnutrition; ^a^ for example, fat free mass index, by dual-energy absorptiometry or corresponding standards using other body composition methods like bioelectrical impedance analysis, computed tomography (CT) or magnetic resonance imaging (MRI). When not available or by regional preference, physical examination or standard anthropometric measures like mid-arm muscle or calf circumference may be used. Thresholds for reduced muscle mass need to be adapted to race (Asia). Functional assessments like hand-grip strength may be considered as a supportive measure. ^b^ Consider gastrointestinal symptoms as supportive indicators that can impair food intake or absorption (e.g., dysphagia, nausea, vomiting, diarrhoea, constipation or abdominal pain). Use clinical judgement to discern severity based upon the degree to which intake or absorption are impaired. Symptom intensity, frequency and duration should be noted. ^c^ Reduced assimilation of food/nutrients is associated with malabsorptive disorders like short bowel syndrome, pancreatic insufficiency and after bariatric surgery. It is also associated with disorders like esophageal stricture, gastroparesis and intestinal pseudo-obstruction. Malabsorption is a clinical diagnosis that manifests as chronic diarrhoea or steatorrhoea. Malabsorption in those with ostomies is evidenced by elevated volumes of output. Use clinical judgement or additional evaluation to discern severity based upon frequency, duration and quantitation of faecal fat and/or volume of losses. ^d^ Acute disease/injury-related. Severe inflammation is likely to be associated with major infection, burns, trauma or closed head injury. Other acute disease/injury-related conditions are likely to be associated with mild-moderate inflammation. ^e^ Chronic disease-related conditions. Severe inflammation is not generally associated with chronic disease conditions. Chronic or recurrent mild-moderate inflammation is likely to be associated with malignant disease, chronic obstructive pulmonary disease, congestive heart failure, chronic renal disease or any disease with chronic or recurrent inflammation. Note that transient inflammation of a mild degree does not meet the threshold for this etiologic criterion. ^f^ C-reactive protein may be used as a supportive laboratory measure.

**Table 2:** Patient characteristics of 246 patients with cancer attending an ambulatory cancer care centre.

	*n* (%)
**Age ^1^**	61.9 ± 13.1
**Gender**	
Male	64 (26)
Female	182 (74)
**BMI (WHO categories) ^2^**	
Underweight (≤18.5 kg/m^2^ for <65 years, <24 for ≥65 years)	30 (14)
Healthy weight (18.5–25 kg/m^2^ for <65 years, 24–31 for ≥65 years)	102 (47)
Overweight (≥25–30 kg/m^2^ for <65 years, >31 for ≥65 years)	53 (24)
Obese (≥30 kg/m^2^ for <65 years)	33 (15)
**Type of cancer**	
Respiratory	21 (9)
Urogenital	15 (6)
Head and neck	1 (1)
Gynaecology	32 (13)
Breast	110 (45)
Haematology	21 (9)
Colorectal	27 (11)
Upper GI	11 (5)
Melanoma	7 (3)
Unknown primary	1 (1)
**Emetogenicity of current cancer treatment ^3^**	
Low	125 (53)
Moderate	64 (27)
High	47 (20)

^1^ Average ± SD, ^2^ *n* = 218, ^3^ *n* = 236. BMI = body mass index; WHO = World Health Organization.

**Table 3:** Sensitivity and specificity of nutritional indices of malnutrition risk and malnutrition as determined by reference standard Patient-Generated Subjective Global Assessment.

	Sensitivity (%)	Specificity (%)	PPV (%)	NPV (%)	Kappa
**Malnutrition Risk**					
PG-SGA SF (≥3)	94	62	31	98	0.311
PG-SGA SF (≥4)	92	71	37	98	0.387
PG-SGA SF (≥5)	89	80	45	98	0.493
PG-SGA SF (≥3) and HGS < 10th percentile	21	95	44	87	0.208
PG-SGA SF (≥4) and HGS < 10th percentile	21	96	50	87	0.229
PG-SGA SF (≥5) and HGS < 10th percentile	21	96	50	87	0.229
**Malnutrition**					
GLIM criteria (moderate, severe or both)	76	73	34	94	0.323
GLIM criteria and HGS < 10th percentile	19	96	43	87	0.186

PG-SGA SF Patient-Generated Subjective Global Assessment Short Form; HGS handgrip strength; GLIM Global Leadership Initiative on Malnutrition; PPV positive predictive value; NPV negative predictive value.

**Table 4:** Malnutrition risk as determined by PG-SGA SF.

	At Risk Malnutrition *n* (%)
PG-SGA SF (≥1)	181 (79.4)
PG-SGA SF (≥2)	131 (57)
PG-SGA SF (≥3)	108 (47)
PG-SGA SF (≥4)	90 (39)
PG-SGA SF (≥5)	71 (31)
PG-SGA SF (≥6)	52 (23)
PG-SGA SF (≥7)	41 (18)

PG-SGA SF Patient-Generated Subjective Global Assessment Short Form.

**Table 5:** Patient experience completing PG-SGA SF.

	*n*	*n* (%)
**Questionnaires**	105	
Completed		100 (95%)
Not completed		4 (4%)
Partially completed		1 (1%)
**The instructions to complete the self-completed tool were clear**	101	
Strongly agree		47 (47%)
Agree		52 (52%)
Neutral		1 (1%)
Disagree		1 (1%)
Strongly disagree		0
**I understood the questions**	101	
Strongly agree		52 (52%)
Agree		46 (46%)
Neutral		2 (2%)
Disagree		1 (1%)
Strongly disagree		0
**Please estimate the time taken to complete the questions**	100	
Less than 3 min		61 (61%)
3–5 min		36 (36%)
More than 5 min		3 (3%)
**The questions took too much time to complete**	100	
Strongly agree		6 (6%)
Agree		6 (6%)
Neutral		7 (7%)
Disagree		47 (47%)
Strongly disagree		34 (34%)

**Table 6:** Associations between malnutrition diagnoses and 1-year mortality in patients with cancer attending an ambulatory cancer care centre.

Malnutrition Diagnosis	Model 1 ^1^ HR (95% CI)	Model 2 ^1^ HR (95% CI)	Model 3 ^1^ HR (95% CI)
MST ≥ 2	2.554 (1.296; 5.032)	3.021 (1.339; 6.817)	3.392 (1.463; 7.865) *p* = 0.004
PG-SGA B or C	7.128 (2.982; 17.034)	7.305 (2.822; 18.908)	10.373 (3.752; 28.681) *p* < 0.001
GLIM-malnutrition	1.951 (0.930; 4.092)	2.186 (0.982; 4.867)	2.238 (1.004; 4.991) *p* = 0.049
GLIM-malnutrition + handgrip strength < 10th percentile	2.952 (0.856; 10.178)	3.237 (0.882; 11.884)	4.136 (1.009; 16.958) *p* = 0.049
GLIM: moderate malnutrition	1.053 (0.481; 2.304)	1.165 (0.518; 2.620)	1.166 (0.522; 2.606) *p* = 0.708
GLIM: moderate malnutrition + handgrip strength < 10th percentile	4.048 (0.498; 32.928)	3.201 (0.377; 27.205)	3.642 (0.410; 32.349) *p* = 0.246
GLIM: severe malnutrition	2.631 (1.187; 5.831)	2.575 (1.100; 6.031)	2.890 (1.193; 7.001) *p* = 0.019
GLIM: severe malnutrition + handgrip strength < 10th percentile	2.908 (0.849; 9.963)	3.102 (0.854; 11.270)	3.796 (0.948; 15.200) *p* = 0.059
PG-SA SF ≥ 3	3.091 (1.488; 6.422)	3.184 (1.436; 7.060)	3.099 (1.372; 7.000) *p* = 0.006
PG-SA SF ≥ 3 + handgrip strength < 10th percentile	1.477 (0.601; 3.632)	1.547 (0.569; 4.210)	1.570 (0.572; 4.310) *p* = 0.381
PG-SA SF ≥ 5	3.195 (1.560; 6.543)	3.436 (1.535; 7.692)	3.512 (1.483; 8.318) *p* = 0.004
PG-SA SF ≥ 5 + handgrip strength < 10th percentile	1.477 (0.601; 3.632)	1.547 (0.569; 4.210)	1.570 (0.572; 4.310) *p* = 0.381
Handgrip strength < 10th percentile	1.713 (0.735; 3.995)	1.913 (0.721; 5.076)	1.958 (0.729; 5.259) *p* = 0.183

HR hazard ratio; CI confidence interval. ^1^ Dependent variable: 1-y mortality, independent variables: Model 1: malnutrition diagnosis (0/1), Model 2: malnutrition diagnosis (0/1), gender (female vs. male), age (≥65 vs. <65 y), BMI (≥30 kg/m^2^ vs. <30 kg/m^2^), Model 3: malnutrition diagnosis (0/1), gender (female vs. male), age (>65 vs. ≤65 y), BMI (≥30 kg/m^2^ vs. <30 kg/m^2^), breast cancer vs. other types of cancer.
